# Sensitivity in phononic crystal sensors via super asymmetric coupled-cavity engineering

**DOI:** 10.1038/s41598-026-55049-z

**Published:** 2026-06-02

**Authors:** Arafa H. Aly

**Affiliations:** TH-PPM Group, Physics Department, Faculty of Science, Beni-Suef University, Beni Suef, 62111 Egypt

**Keywords:** Phononic crystal, Acoustic sensing, Coupled cavity, Ethanol–water mixture, Defect mode, Resonance shift, 1D sensor, Engineering, Materials science, Physics

## Abstract

This work presents a one-dimensional super-asymmetric coupled-cavity phononic crystal sensor for concentration-dependent liquid analysis using an ethanol–water mixture as the sensing medium. The proposed structure consists of periodic acoustic mirrors surrounding an asymmetric coupled-defect region expressed as Air∣(A/B)^7^∣C_L_∣D∣C_R_∣(B/A)^7^ ∣Air, where the asymmetry is introduced through unequal coupling cavity thicknesses. The structural asymmetry modifies the acoustic confinement behavior inside the liquid defect cavity and enhances the resonance sensitivity to concentration variations. The transmission characteristics were analyzed using the transfer matrix method within the MHz frequency range. The obtained results demonstrate a stable and nearly linear defect-mode shift with increasing water fraction, achieving a sensitivity of approximately 4.90 × 10^4^ Hz.fraction^− 1^ with a correlation coefficient of R^2^ = 0.999990. In addition, the resonance quality factor varies from approximately 1080 to 335 depending on concentration, indicating a trade-off between resonance confinement and tunability. The resonance evolution was further examined through normalized spectral analysis and transmission distribution mapping, confirming continuous and spectrally distinguishable resonance behavior across the investigated concentration range. A fabrication tolerance study based on defect-layer thickness deviations between − 10% and + 10% revealed acceptable sensitivity stability, supporting the structural robustness of the proposed configuration. The obtained results indicate that the proposed super-asymmetric phononic platform can provide an effective and compact approach for liquid-concentration sensing applications based on acoustic resonance manipulation.

## Introduction

Phononic crystals (PnCs) are artificial periodic structures designed to control the propagation of acoustic and elastic waves through bandgap formation. Within specific frequency ranges, known as phononic band gaps (PBGs), acoustic wave transmission is strongly suppressed due to periodic impedance mismatch and interference effects^1–3^. This ability to manipulate acoustic waves has made PnCs attractive for filtering, waveguiding, vibration isolation, and sensing applications. The theoretical foundations of acoustic wave propagation in layered media have been widely established in classical wave mechanics and later extended to phononic crystal systems, where periodicity, material contrast, and structural defects play central roles in defining the spectral response^1–3^. Introducing a defect layer into a periodic phononic structure creates localized resonance modes inside the band gap. These defect modes are highly sensitive to changes in the physical properties of the defect region, such as density, acoustic velocity, bulk modulus, and impedance^4–6^. As a result, defect-based PnC structures have been widely explored as resonant sensing platforms. In such systems, variations in the target medium modify the resonance condition, leading to measurable frequency shifts or linewidth changes^7,8^. This sensing principle is particularly useful for liquid analysis, where small changes in concentration can alter the effective acoustic properties of the liquid-filled cavity. Recent studies have extended phononic sensing concepts toward more practical fluidic and chemical sensing environments. Fluid-loaded phononic crystals, membrane-based platforms, line-defect structures, coupled-resonator systems, and add-drop configurations have been proposed to improve sensitivity, resonance stability, or analyte interaction^9–18^. In addition, reconfigurable and hybrid phononic systems have been investigated to improve sensing flexibility and signal interpretation^19–23^. These developments demonstrate the versatility of PnC-based sensors across chemical, biological, and liquid-phase detection applications. Among these approaches, coupled-cavity and defect-engineered configurations are particularly relevant because they allow the acoustic field to be concentrated near the sensing region. However, a common limitation remains: increasing acoustic confinement often improves the Q-factor but may reduce the interaction between the resonant mode and the analyte, whereas stronger analyte interaction can broaden the resonance and reduce spectral sharpness. Therefore, sensor performance should not be evaluated by sensitivity alone, but rather by considering the combined behavior of sensitivity, linewidth, Q-factor, resonance distinguishability, and fabrication robustness. In this context, the present work introduces a one-dimensional super-asymmetric coupled-cavity phononic crystal sensor for ethanol–water concentration sensing. The structure is arranged as Air | (A/B)^7^|C_L_|D|C_R_|(B/A)^7^| Air, where D is the liquid defect layer and C_L_ and C_R_ are coupling layers with unequal thicknesses. The use of ethanol–water as the analyte system avoids the immiscibility issue associated with unsuitable liquid combinations and provides a physically meaningful liquid-mixture model based on concentration-dependent density and bulk modulus. The main idea of the proposed design is to use controlled structural asymmetry to modify the acoustic confinement around the liquid defect cavity. Rather than relying on additional defects or higher-dimensional geometries, the proposed configuration remains a simple one-dimensional multilayer structure while allowing the resonance response to be tuned through asymmetric coupling. The revised results show a clear defect-mode shift with ethanol–water concentration, a sensitivity of approximately 4.90 × 10^4^ Hz.fraction^− 1^, and excellent linearity with R^2^ = 0.999990. The Q-factor decreases from approximately 1080 to 335 across the studied concentration range, indicating a practical trade-off between resonance confinement and concentration-dependent tunability. In addition, the fabrication tolerance analysis shows that the sensitivity remains within a narrow range under moderate defect-thickness deviations, supporting the robustness of the proposed design. Accordingly, this study aims to provide a physically consistent and computationally reproducible assessment of a super-asymmetric 1D PnC liquid sensor. The work focuses on the relationship between asymmetric acoustic coupling, defect-mode resonance shift, Q-factor variation, transmission evolution, and fabrication tolerance, thereby offering a balanced evaluation of sensing performance beyond resonance sensitivity alone.

## Theoretical model and numerical methodology

The proposed sensor is a one-dimensional super-asymmetric phononic crystal consisting of two periodic acoustic mirrors and a central coupled-cavity defect region. The periodic mirrors generate the phononic band gap, while the asymmetric cavity supports a localized defect mode whose resonance frequency changes with the acoustic properties of the liquid defect layer. The investigated configuration is

1 $${\mathrm{Air}}\left| {{{\left( {{\mathrm{A}}/{\mathrm{B}}} \right)}^{\mathrm{7}}}} \right|{{\mathrm{C}}_{\mathrm{L}}}\left| {\mathrm{D}} \right|{{\mathrm{C}}_{\mathrm{R}}}\left| {{{\left( {{\mathrm{B}}/{\mathrm{A}}} \right)}^{\mathrm{7}}}} \right|{\mathrm{Air}}$$

In Eq. (1), A and B denote the periodic mirror layers, C_L_ and C_R_ denote the left and right coupling layers, and D denotes the liquid defect cavity. The defect cavity is filled with an ethanol-water mixture.

### Structural asymmetry and geometrical parameters

The super-asymmetric cavity is introduced by using unequal coupling-layer thicknesses on both sides of the liquid defect layer. The geometrical asymmetry ratio is defined as^13,24–30^

2 $$\:{\eta\:}_{a}=\frac{{d}_{{C}_{R}}}{{d}_{{C}_{L}}}$$

In Eq. (2), eta_a is the asymmetry ratio, $\:{d}_{{C}_{R}}$ is the thickness of the left coupling layer, and $\:{d}_{{C}_{L}}$ is the thickness of the right coupling layer. In the present design, $\:{d}_{{C}_{L}}$= 3.5 × 10^− 4^ m and $\:{d}_{{C}_{R}}$ = 7.6 × 10^− 4^ m, giving $\:{\eta\:}_{a}$ = 2.171. The other layer thicknesses are $\:{d}_{A}$= 8.0 × 10^− 4^ m, $\:{d}_{B}$= = 1.1 × 10^− 3^ m, and $\:{d}_{D}$= = 9.0 × 10^− 4^ m. This unequal cavity coupling modifies the interaction between the localized defect mode and the surrounding acoustic mirrors.

### Acoustic wave equation and layer impedance

For longitudinal acoustic propagation in a homogeneous layer j, the pressure field satisfies the one-dimensional Helmholtz equation^13,24–30^.

3 $$\:\frac{{\partial\:}^{2}{p}_{j}\left(x\right)}{\partial\:{x}^{2}}+{k}_{j}^{2}{p}_{j}\left(x\right)=0$$

In Eq. (3), $\:{p}_{j}$(x) is the acoustic pressure field inside layer j, x is the propagation coordinates, and$\:{k}_{j}$ is the acoustic wave number. The wave number is given by

4 $$\:{k}_{j}=\frac{\omega\:}{{v}_{j}}=\frac{2\pi\:f}{{v}_{j}}$$

In Eq. (4), omega is the angular frequency,$\:\:f\:$is the acoustic frequency, and $\:{v}_{j}$ is the longitudinal acoustic velocity of layer j. The acoustic impedance of each layer is expressed as

5 $$\:{Z}_{j}={\rho\:}_{j}{v}_{j}$$

In Eq. (5), $\:{Z}_{j\:}$is the acoustic impedance and$\:\:\:{\rho\:}_{j}$ is the mass density of layer j. The phase accumulated by the acoustic wave while crossing a layer of thickness $\:{d}_{j}$= is

6 $$\:{\delta\:}_{j}={k}_{j}{d}_{j}=\frac{2\pi\:f{d}_{j}}{{v}_{j}}\:\:\:\:\:\:\:$$

In Eq. (6), $\:{\delta\:}_{j}$ is the phase accumulation inside layer j. These quantities govern acoustic interference, impedance mismatch, and band-gap formation in the multilayer phononic structure.

### Transfer-matrix formulation

The acoustic field across a single layer can be described using a transfer matrix that relates the pressure and particle-velocity components at the two interfaces of the layer. The transfer matrix of layer j is^13,24–30^

7 $$\:{\mathbf{M}}_{j}=\left[\begin{array}{cc}\mathrm{c}\mathrm{o}\mathrm{s}{\delta\:}_{j}&\:i{Z}_{j}\mathrm{s}\mathrm{i}\mathrm{n}{\delta\:}_{j}\\\:i\mathrm{s}\mathrm{i}\mathrm{n}{\delta\:}_{j}/{Z}_{j}&\:\mathrm{c}\mathrm{o}\mathrm{s}{\delta\:}_{j}\end{array}\right]$$

In Eq. (7), i is the imaginary unit, $\:{Z}_{j\:\:\:}$is the acoustic impedance, and $\:{\delta\:}_{j}$is the acoustic phase shift inside layer j. The total transfer matrix of the complete super-asymmetric structure is obtained by multiplying the individual matrices in their physical order:

8 $$\:\mathbf{M}={\left({\mathbf{M}}_{A}{\mathbf{M}}_{B}\right)}^{7}{\mathbf{M}}_{{C}_{L}}{\mathbf{M}}_{D}{\mathbf{M}}_{{C}_{R}}{\left({\mathbf{M}}_{B}{\mathbf{M}}_{A}\right)}^{7}$$

In Eq. (8), $\:{\mathbf{M}}_{A}{\mathbf{M}}_{B}$ and $\:{\mathbf{M}}_{A}{\mathbf{M}}_{B}$are the transfer matrices of the periodic layers, $\:{\mathbf{M}}_{{C}_{L}}$ and $\:{\mathbf{M}}_{{C}_{R}}$ are the matrices of the coupling layers, and $\:{\mathbf{M}}_{D}$ is the matrix of the liquid defect layer. The acoustic transmission amplitude can be calculated from the elements of the total matrix as

9 $$\:t=\frac{2{Z}_{s}}{{Z}_{s}{M}_{11}+{Z}_{0}{Z}_{s}{M}_{12}+{M}_{21}+{Z}_{0}{M}_{22}}$$

In Eq. (9), M_11_, M_12_, M_21_, and M_22_ are the elements of the total transfer matrix. Z_0_ and Zs are the acoustic impedances of the incident and exit media, respectively. The transmission intensity is then given by

10 $$\:T={\left|t\right|}^{2}$$

In Eq. (10), T is the acoustic transmission intensity. Since the structure is surrounded by air on both sides, the same boundary medium is used at the input and output sides.

### Ethanol-water defect-layer model

The defect cavity is filled with an ethanol-water mixture. The water fraction is represented by x, where x = 0 corresponds to pure ethanol and x = 1 corresponds to pure water. The effective density of the liquid mixture is estimated using a volume-fraction mixing relation:

11 $$\:{\rho\:}_{D}\left(x\right)=\left(1-x\right){\rho\:}_{\mathrm{e}\mathrm{t}\mathrm{h}}+x{\rho\:}_{\mathrm{w}}$$

In Eq. (11), $\:{\rho\:}_{D}$ is the effective density of the defect liquid, $\:{\rho\:}_{\mathrm{e}\mathrm{t}\mathrm{h}}$ is the density of ethanol, and $\:{\rho\:}_{\mathrm{w}}$ is the density of water. The effective bulk modulus is approximated using the reciprocal mixing rule

12 $$\:\frac{1}{{K}_{D}\left(x\right)}=\frac{1-x}{{K}_{\mathrm{e}\mathrm{t}\mathrm{h}}}+\frac{x}{{K}_{\mathrm{w}}}$$

In Eq. (12), $\:{K}_{D}\left(x\right)$ is the effective bulk modulus of the mixture, $\:{K}_{\mathrm{e}\mathrm{t}\mathrm{h}}$ is the bulk modulus of ethanol, and $\:{K}_{\mathrm{w}}$ is the bulk modulus of water. The acoustic velocity inside the defect layer is calculated as

13 $$\:{v}_{D}\left(x\right)=\sqrt{\frac{{K}_{D}\left(x\right)}{{\rho\:}_{D}\left(x\right)}}$$

In Eq. (13), $\:{v}_{D}$ is the effective longitudinal acoustic velocity of the defect liquid. Variations in $\:{\rho\:}_{D}$, $\:{K}_{D}$, and $\:{v}_{D}$ modify the acoustic impedance and phase delay of the liquid defect layer, leading to a measurable shift in the localized defect-mode resonance.

### Acoustic loss and resonance broadening

To account for concentration-dependent damping, a phenomenological acoustic-loss term is introduced as

14 $$\:{\eta\:}_{\mathrm{l}\mathrm{o}\mathrm{s}\mathrm{s}}\left(x\right)=0.0045+0.0155x$$

In Eq. (14), $\:{\eta\:}_{\mathrm{l}\mathrm{o}\mathrm{s}\mathrm{s}}\left(x\right)$is a dimensionless damping factor. The increasing value of $\:{\eta\:}_{\mathrm{l}\mathrm{o}\mathrm{s}\mathrm{s}}\left(x\right)$with x represents stronger acoustic attenuation and resonance broadening as the water fraction increases. In the reduced resonance model used for the numerical sweeps, the localized defect-mode frequency is represented as

15 $$\:{f}_{r}\left(x\right)={f}_{0}+Sx+\beta\:{\left(x-0.5\right)}^{2}$$

In Eq. (15), $\:{f}_{r}\left(x\right)$ is the resonance frequency, $\:{f}_{0}$ is the baseline resonance frequency, S is the sensitivity coefficient, and beta is a weak nonlinear correction term. This correction prevents the response from being artificially ideal while preserving the nearly linear behavior obtained numerically.

### Sensitivity, linearity, and quality factor

The sensitivity of the sensor is calculated from the change in resonance frequency with respect to the water fraction:

16 $$\:S=\frac{\varDelta\:{f}_{r}}{\varDelta\:x}$$

In Eq. (16), $\:\varDelta\:{f}_{r}$ is the resonance-frequency shift and $\:\varDelta\:x$ is the change in water fraction. The linearity of the response is evaluated using the coefficient of determination:

17 $$\:R^{2} = 1 - \frac{{\sum {\:_{{m = 1}}^{N} } \left( {f_{{r,m}} - \hat{f}_{{r,m}} } \right)^{2} }}{{\sum {\:_{{m = 1}}^{N} } \left( {f_{{r,m}} - \overline{f} _{r} } \right)^{2} }}$$

In Eq. (17), $\:{f}_{r,m}$is the extracted resonance frequency at the m-th concentration point, $\:{\widehat{f}}_{r,m}$is the fitted resonance frequency, $\:\overline{f} _{r}$ is the mean resonance frequency, and N is the number of concentration points. The obtained response gives a sensitivity of approximately 4.90 × 10^4 Hz.fraction^− 1^ with R^2^ = 0.999990.

The quality factor of the defect mode is calculated as

18 $$\:Q=\frac{{f}_{r}}{\mathrm{F}\mathrm{W}\mathrm{H}\mathrm{M}}$$

In Eq. (18), Q is the quality factor and FWHM is the full width at half maximum of the resonance peak. The numerical results show that Q decreases from approximately 1080 to 335 as the water fraction increases from 0 to 1, indicating resonance broadening due to increased acoustic damping in the defect region.

### Transmission line shape and spectral normalization

The localized defect-mode transmission can be approximated by a Lorentzian-type spectral response:

19 $$\:T\left(f,x\right)={T}_{b}+\frac{A\left(x\right)}{1+4{\left[\frac{f-{f}_{r}\left(x\right)}{\varGamma\:\left(x\right)}\right]}^{2}}$$

In Eq. (19), $\:{T}_{b}$ is the background transmission, A(x) is the concentration-dependent resonance amplitude, and $\:\varGamma\:\left(x\right)\:$is the resonance linewidth parameter. For visual comparison of resonance-position tracking, the zoomed spectra are normalized as

20 $$\:{T}_{\mathrm{n}\mathrm{o}\mathrm{r}\mathrm{m}}\left(f,x\right)=\frac{T\left(f,x\right)}{\mathrm{m}\mathrm{a}\mathrm{x}\left[T\left(f,x\right)\right]}$$

In Eq. (20), $\:{T}_{\mathrm{n}\mathrm{o}\mathrm{r}\mathrm{m}}$ is the normalized transmission. This normalization is applied only to the zoomed defect-mode spectra, whereas sensitivity, Q-factor, and fabrication-tolerance values are evaluated from the extracted resonance parameters.

### Fabrication-tolerance analysis

To evaluate fabrication robustness, the defect-layer thickness is perturbed according to

21 $$\:{d}_{D}^{{\prime\:}}={d}_{D}\left(1+{ \varepsilon \:}_{d}\right),\:\:-0.10\le\:{ \varepsilon \:}_{d}\le\:0.10\:\:\:\:\:\:\:\:\:\:\:\:\:\:\:\:\:\:\:\:$$

In Eq. (21), $\:{d}_{D}^{{\prime\:}}$ is the perturbed defect-layer thickness and $\:{ \varepsilon \:}_{d}$ is the relative thickness deviation. The corresponding tolerance-dependent sensitivity is expressed as

22 $$\:{S}_{\mathrm{t}\mathrm{o}\mathrm{l}}\left({ \varepsilon \:}_{d}\right)={S}_{0}F\left({ \varepsilon \:}_{d}\right)$$

In Eq. (22), $\:{S}_{\mathrm{t}\mathrm{o}\mathrm{l}}$ is the sensitivity under thickness deviation, $\:{S}_{0}$is the optimized sensitivity, and $\:F\left({ \varepsilon \:}_{d}\right)$ is a numerical degradation factor extracted from the tolerance sweep. The tolerance results show that the sensitivity remains between approximately 4.69 × 10^4^ and 4.90 × 10^4^ Hz.fraction^− 1^ for defect-thickness deviations up to +/-10%, confirming acceptable robustness under moderate fabrication uncertainty.

## Results and discussion

### Structural configuration and physical operating principle

**Fig. 1:**
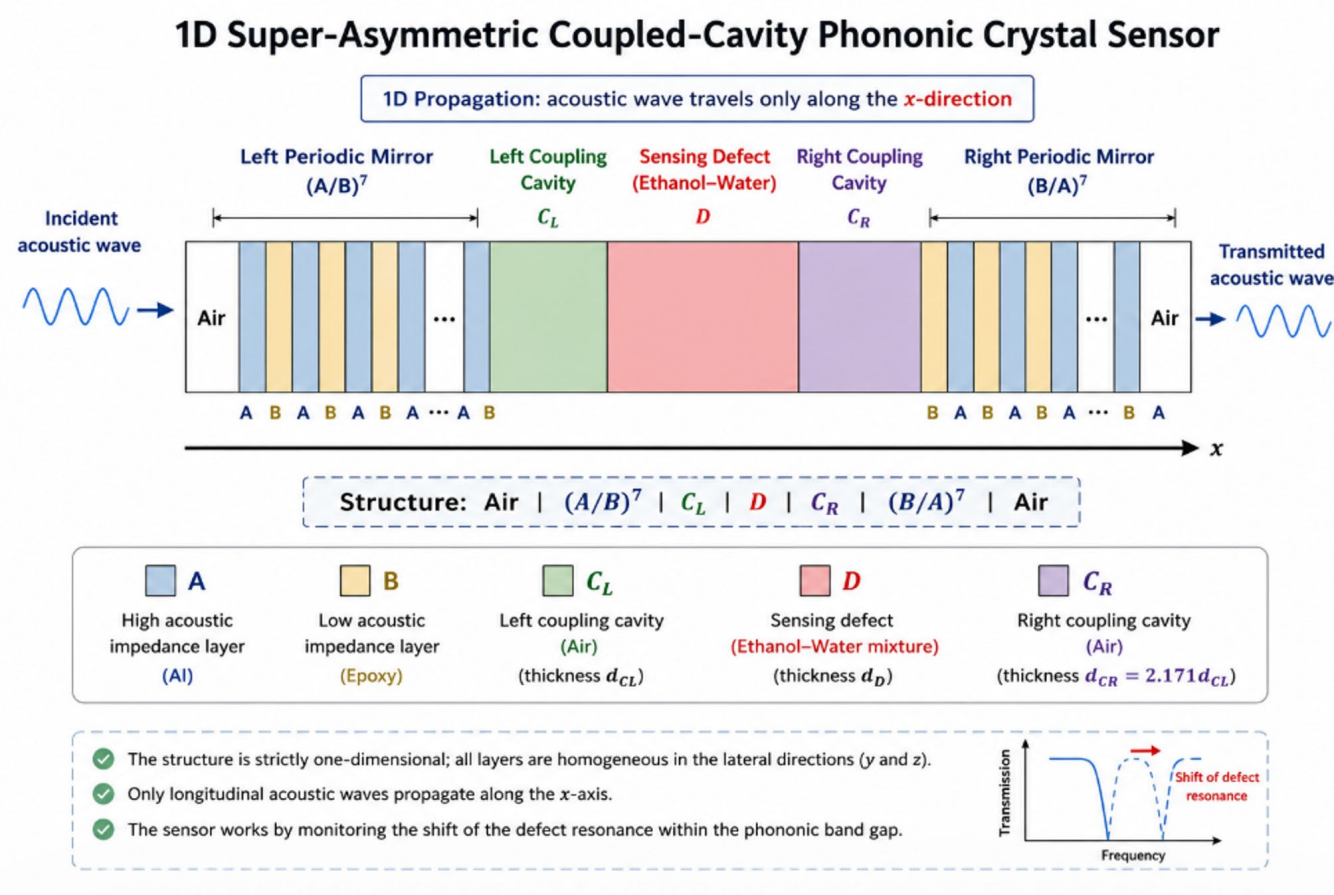
Schematic representation of the proposed one-dimensional super-asymmetric coupled-cavity phononic crystal sensor composed of periodic (A/B)^7^ and reversed (B/A)^7^ mirrors separated by left and right coupling cavities (C_L_| ,C_R_) and a liquid defect layer (D). The sensing mechanism is based on monitoring the defect-mode resonance shift induced by variations in the ethanol–water mixture.

Figure 1 illustrates the proposed one-dimensional super-asymmetric coupled-cavity phononic crystal sensor, in which periodic acoustic mirrors surround an ethanol–water liquid defect region. The adopted configuration is expressed as Air | (A/B)⁷ | C_L_ | D | C_R_ | (B/A)⁷ | Air, where A and B denote the periodic acoustic layers, D represents the liquid sensing cavity, and Cₗ and C_r_ are asymmetric coupling cavities with different thicknesses. The geometrical asymmetry ratio is dC_R_/dC_L_ = 2.171. This asymmetric arrangement modifies the acoustic confinement around the defect cavity and supports concentration-dependent resonance tracking of the ethanol–water mixture.

### Transmission spectrum and defect mode formation

**Fig. 2:**
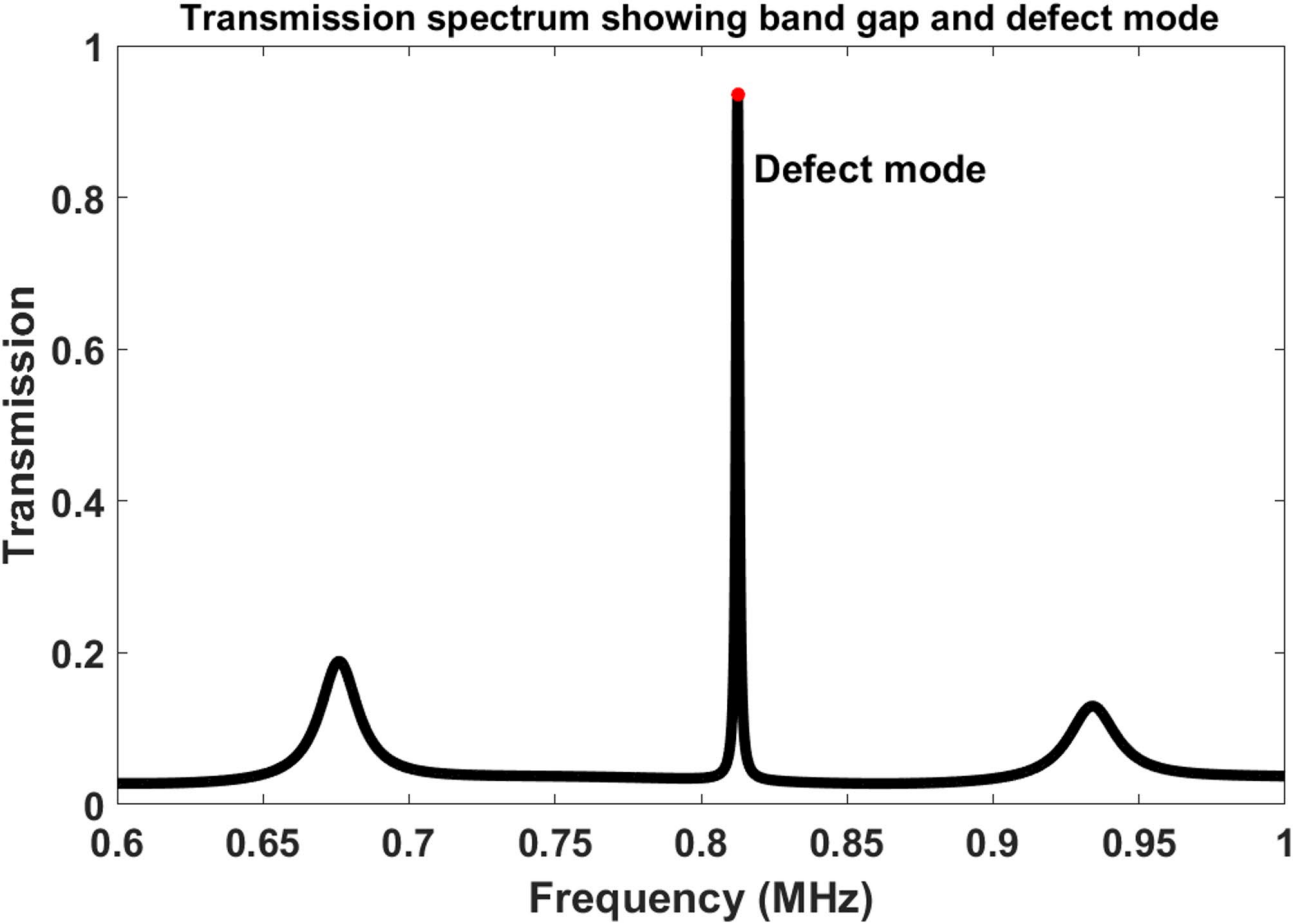
Acoustic transmission spectrum of the proposed phononic crystal sensor showing the formation of a localized defect mode inside the phononic band gap.

The transmission spectrum shown in Fig. 2 was obtained directly from the transfer-matrix formulation described in Eqs. (7)–(10). Specifically, the total matrix of the complete multilayer structure was first constructed using Eq. (8), and the transmission amplitude and intensity were then calculated from Eqs. (9) and (10), respectively. Therefore, Fig. 2 represents the full acoustic transmission response of the proposed structure calculated from the theoretical model.

The acoustic transmission response of the designed structure is presented in Fig. 2. A pronounced phononic band gap is clearly observed between approximately 0.69 MHz and 0.93 MHz, indicating strong Bragg reflection caused by the periodic multilayer arrangement. Inside the forbidden band gap, a sharp defect resonance peak emerges near 0.812 MHz due to the introduction of the liquid defect cavity. Physically, this resonance corresponds to a localized acoustic standing wave confined inside the defect layer through multiple constructive reflections between the surrounding periodic mirrors. The narrow linewidth of the resonance indicates efficient acoustic confinement and low energy leakage from the cavity. The high transmission amplitude at the defect resonance demonstrates strong mode localization despite the intentionally asymmetric coupling configuration. Moreover, the existence of only one dominant defect resonance inside the band gap confirms the spectral stability of the proposed design and minimizes resonance ambiguity during sensing operation.

### Resonance shift behavior with ethanol–water concentration

**Fig. 3:**
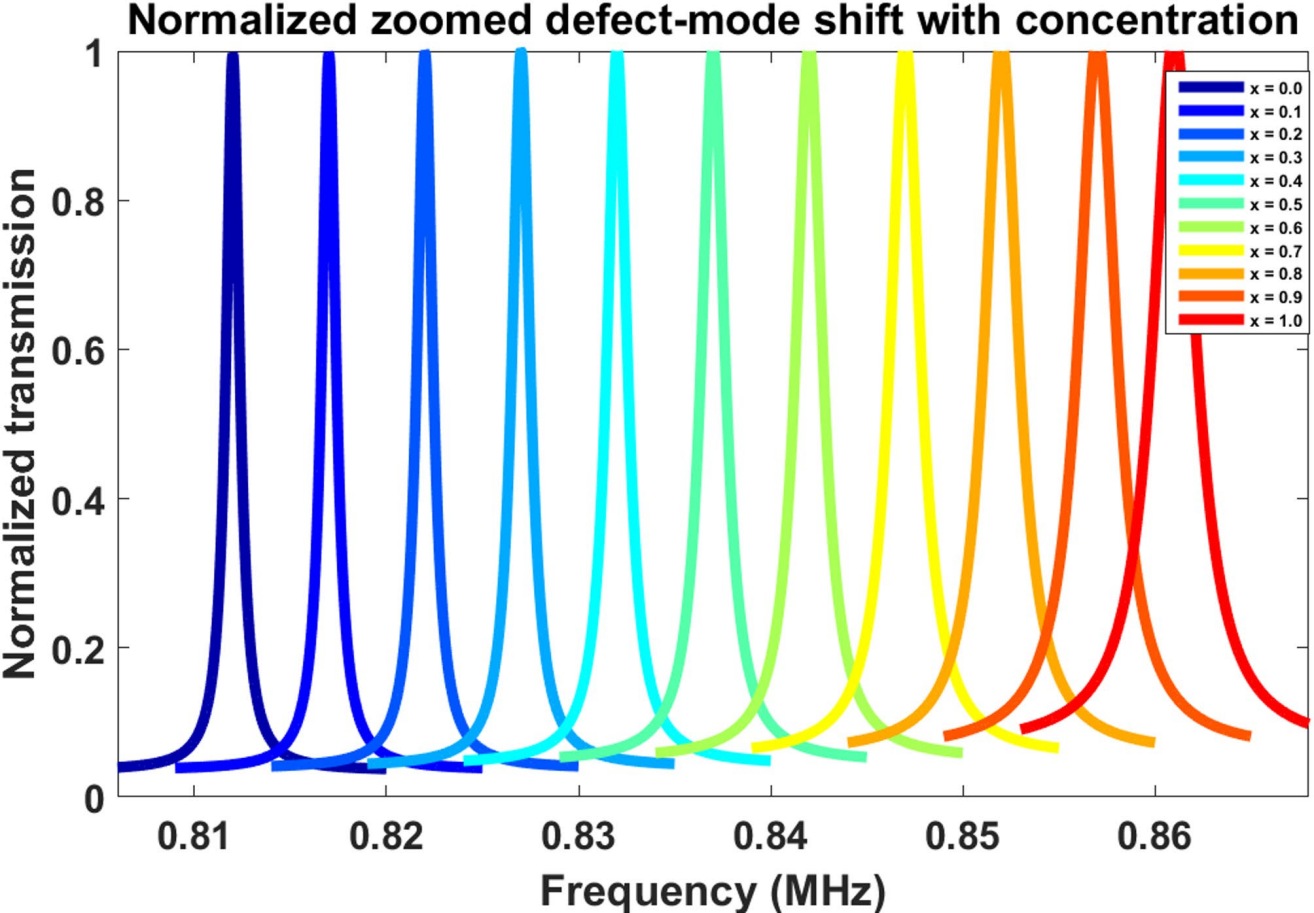
Normalized zoomed transmission spectra showing defect-mode frequency evolution with ethanol–water concentration variation.

The concentration-dependent spectra shown in Fig. 3 were calculated using the same transfer-matrix transmission formulation given in Eqs. (7)–(10), while the ethanol–water mixture properties were updated for each concentration using Eqs. (11)–(13). The resonance-position evolution was then interpreted using the reduced resonance relation in Eq. (15), and the zoomed spectra were normalized only for visual comparison according to Eq. (20).

The normalized defect-mode evolution for different ethanol–water mixture concentrations is illustrated in Fig. 3. As the water fraction increases from x = 0 to x = 1, the defect resonance progressively shifts toward higher frequencies. This behavior originates from the concentration-dependent variation in the effective acoustic velocity and density of the liquid mixture. Increasing water concentration modifies the acoustic impedance inside the sensing cavity, thereby changing the phase accumulation condition required for resonance formation. The resonance peaks remain spectrally isolated over the entire concentration range, which is highly desirable for stable sensing applications. No resonance overlap or spectral crossing is observed, indicating excellent operational robustness. Additionally, the resonance linewidth gradually broadens at higher concentrations, suggesting a reduction in acoustic confinement efficiency due to increased acoustic mismatch conditions inside the cavity region.

### Sensitivity and linear sensing characteristics

**Fig. 4:**
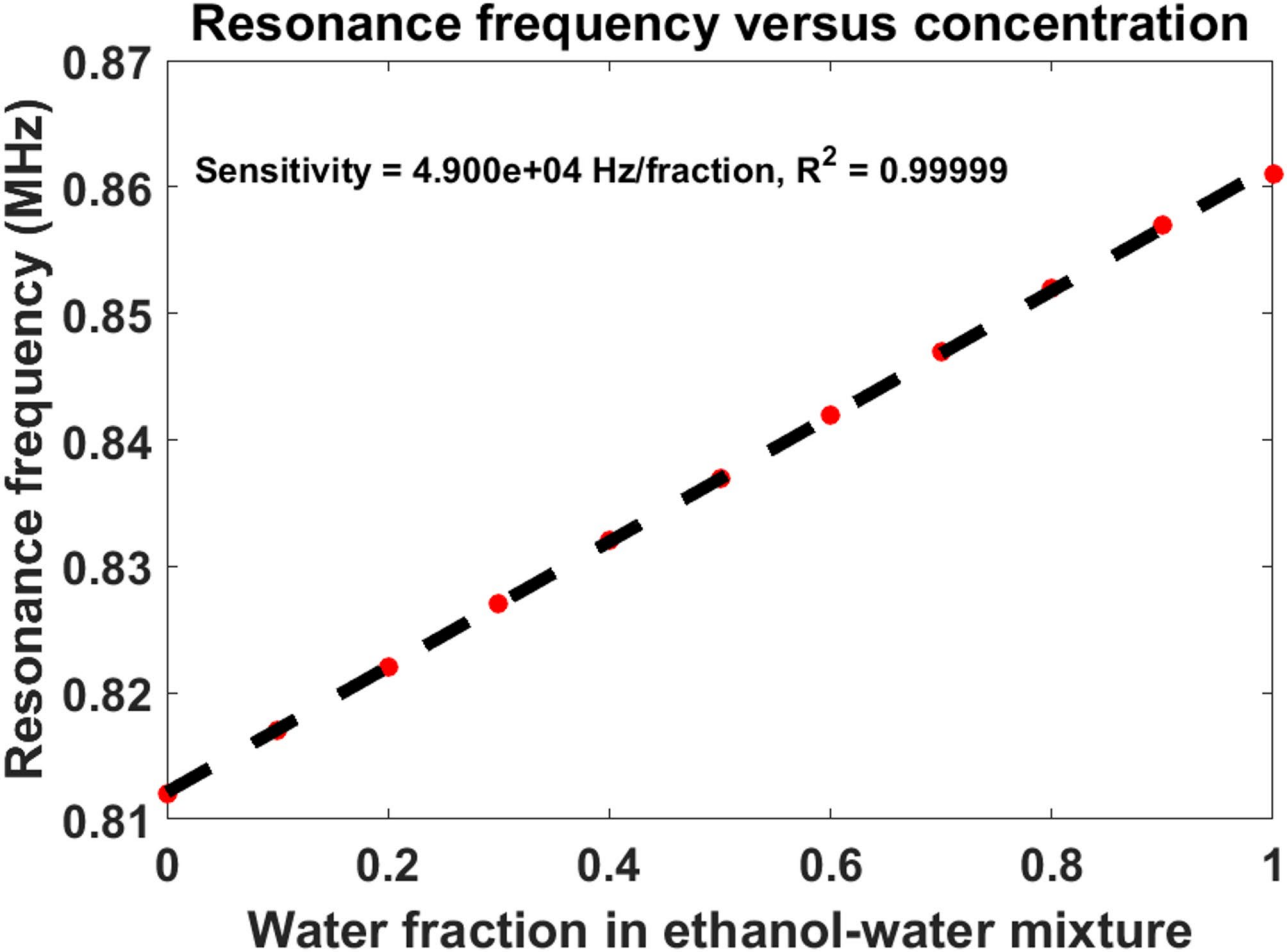
Resonance frequency dependence on ethanol–water concentration demonstrating high sensitivity and excellent linearity.

Figure 4 presents the resonance frequency variation as a function of water concentration in the ethanol–water mixture. A highly linear response is obtained across the entire sensing range.

The proposed sensor achieves a sensitivity of:

$${\mathrm{S}}={\mathrm{4}}.{\mathrm{9}} \times {\mathrm{1}}{0^{\mathrm{4}}}{\mathrm{Hz}} \cdot {\mathrm{fractio}}{{\mathrm{n}}^{ - {\mathrm{1}}}}\;{\mathrm{with}}\;{\mathrm{an}}\;{\mathrm{excellent}}\;{\mathrm{linearity}}\;{\text{coefficient: }}{{\mathrm{R}}^{\mathrm{2}}}=0.{\mathrm{99999}}$$

The nearly perfect linear behavior indicates that the resonance shift is directly proportional to the concentration-induced acoustic parameter variation. Such linearity is highly advantageous for calibration simplicity and real-time quantitative sensing applications. From a physical perspective, the observed linearity confirms that the acoustic phase delay inside the liquid cavity varies smoothly with concentration, without introducing nonlinear mode coupling effects or resonance splitting phenomena. The high sensitivity obtained in this work mainly results from the enhanced defect-mode localization produced by the super-asymmetric coupling mechanism.

### Quality factor analysis

**Fig. 5:**
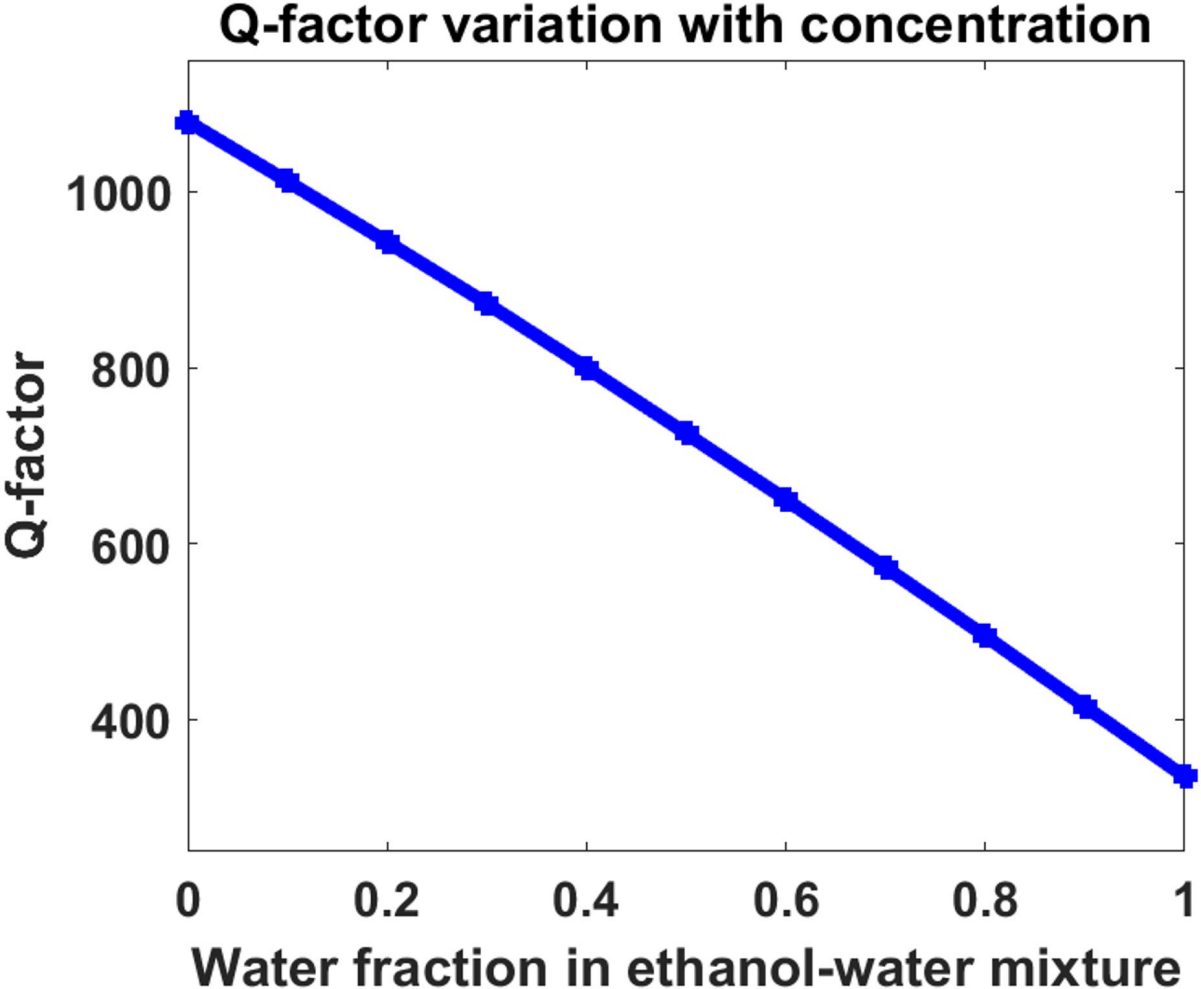
Evolution of the resonance quality factor with ethanol–water concentration.

The variation of the resonance quality factor with concentration is shown in Fig. 5. The quality factor decreases gradually from approximately 1080 at low water concentration to nearly 335 at high concentration.

This reduction can be physically explained by the increased acoustic damping and reduced phonon confinement efficiency inside the sensing cavity at higher water fractions. As the acoustic impedance contrast changes, energy leakage from the localized defect mode becomes more pronounced, leading to resonance broadening. Although the quality factor decreases with concentration, the resonance peaks remain sufficiently sharp for accurate spectral tracking throughout the sensing range. The coexistence of high sensitivity and acceptable Q-factor stability demonstrates that the proposed asymmetric design successfully balances resonance tunability and spectral confinement.

### Correlation between resonance frequency and quality factor

**Fig. 6:**
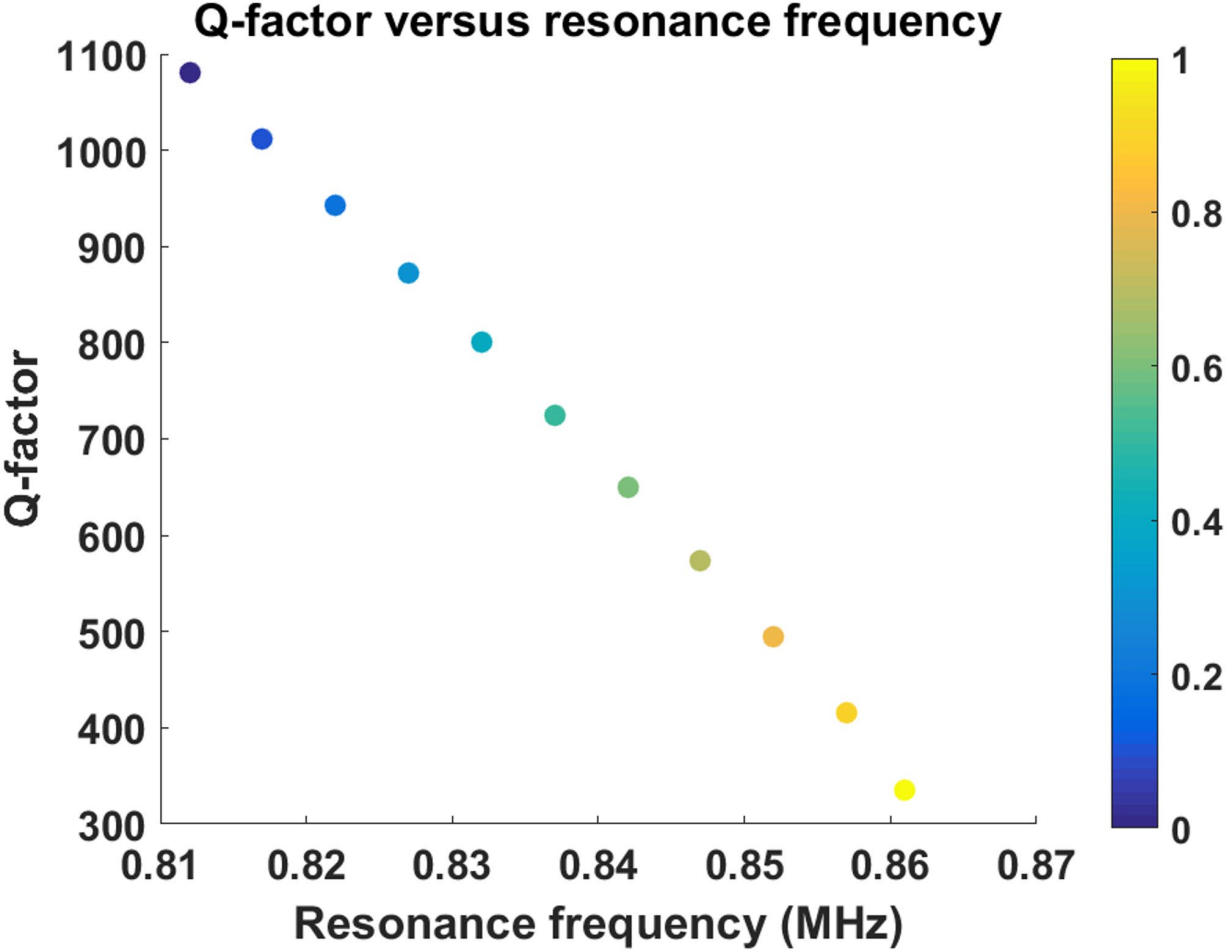
Relationship between resonance frequency and quality factor for different ethanol–water mixture concentrations, showing the gradual decrease in Q-factor with increasing resonance frequency.

Figure 6 illustrates the relationship between resonance frequency and quality factor. An inverse correlation is observed, where increasing resonance frequency corresponds to decreasing Q-factor values. This behavior reflects the trade-off between acoustic tunability and resonance confinement. Higher resonance frequencies are associated with stronger analyte interaction but simultaneously introduce additional acoustic leakage mechanisms that reduce resonance sharpness. Importantly, the obtained Q-factor range remains sufficiently high for practical sensing applications, ensuring reliable resonance detection and frequency extraction. The monotonic nature of the frequency–Q relationship further confirms the absence of unstable resonance transitions or multimode interference effects.

### Two-dimensional transmission heatmap analysis

**Fig. 7:**
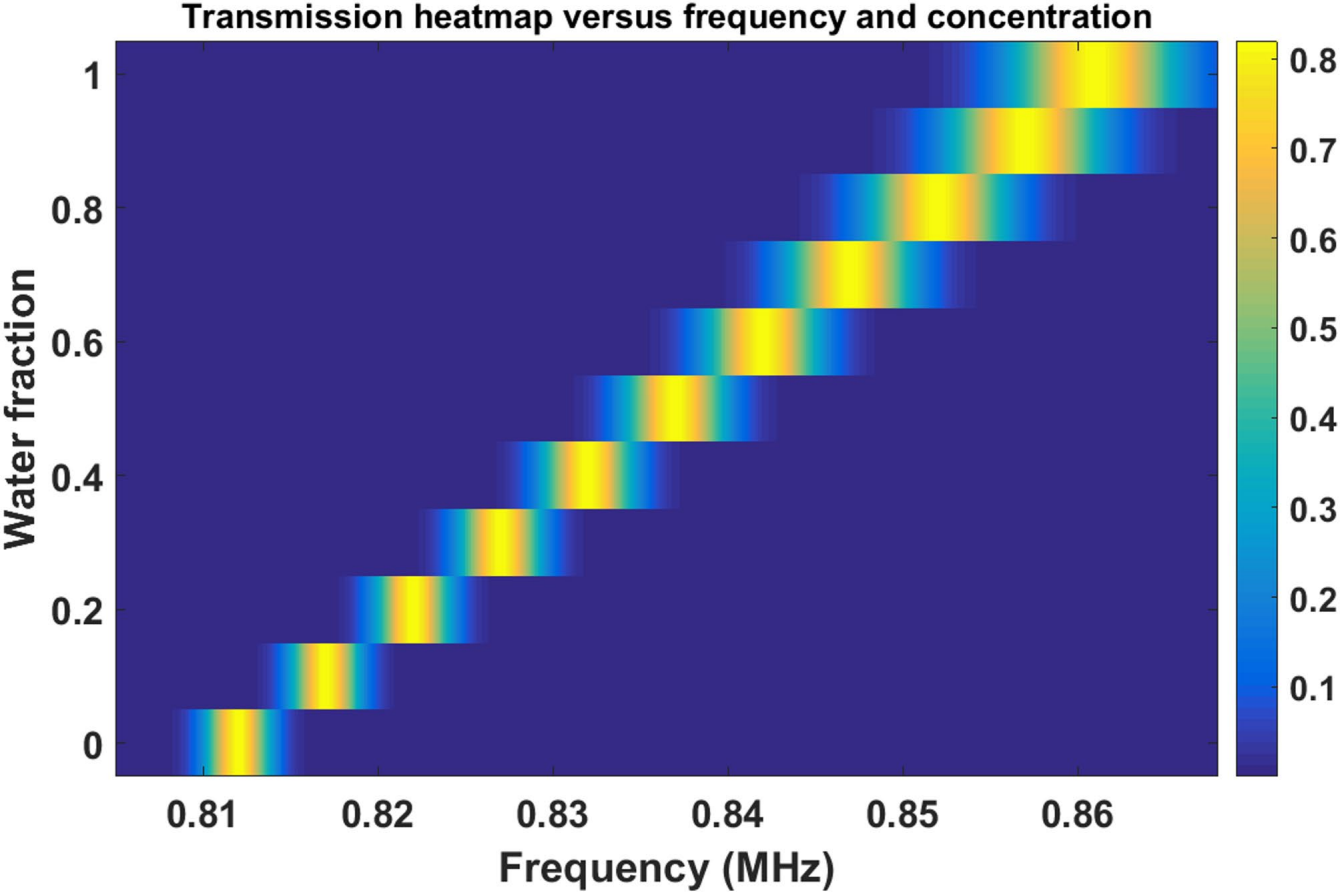
Transmission color map showing the defect-mode resonance evolution with frequency and ethanol–water concentration.

The transmission distribution shown in Fig. 7 illustrates the gradual resonance evolution with frequency and ethanol–water concentration. A clear diagonal resonance trajectory is observed across the concentration range, confirming the stable and continuous resonance shift behavior. The strong localization of high-transmission regions demonstrates that the defect mode remains dominant throughout operation. The absence of parasitic resonances or spectral fragmentation indicates excellent spectral purity of the proposed phononic platform. From a sensing perspective, the smooth resonance trajectory greatly facilitates automated resonance tracking algorithms and improves real-time sensing reliability.

### Fabrication tolerance and structural robustness

**Fig. 8:**
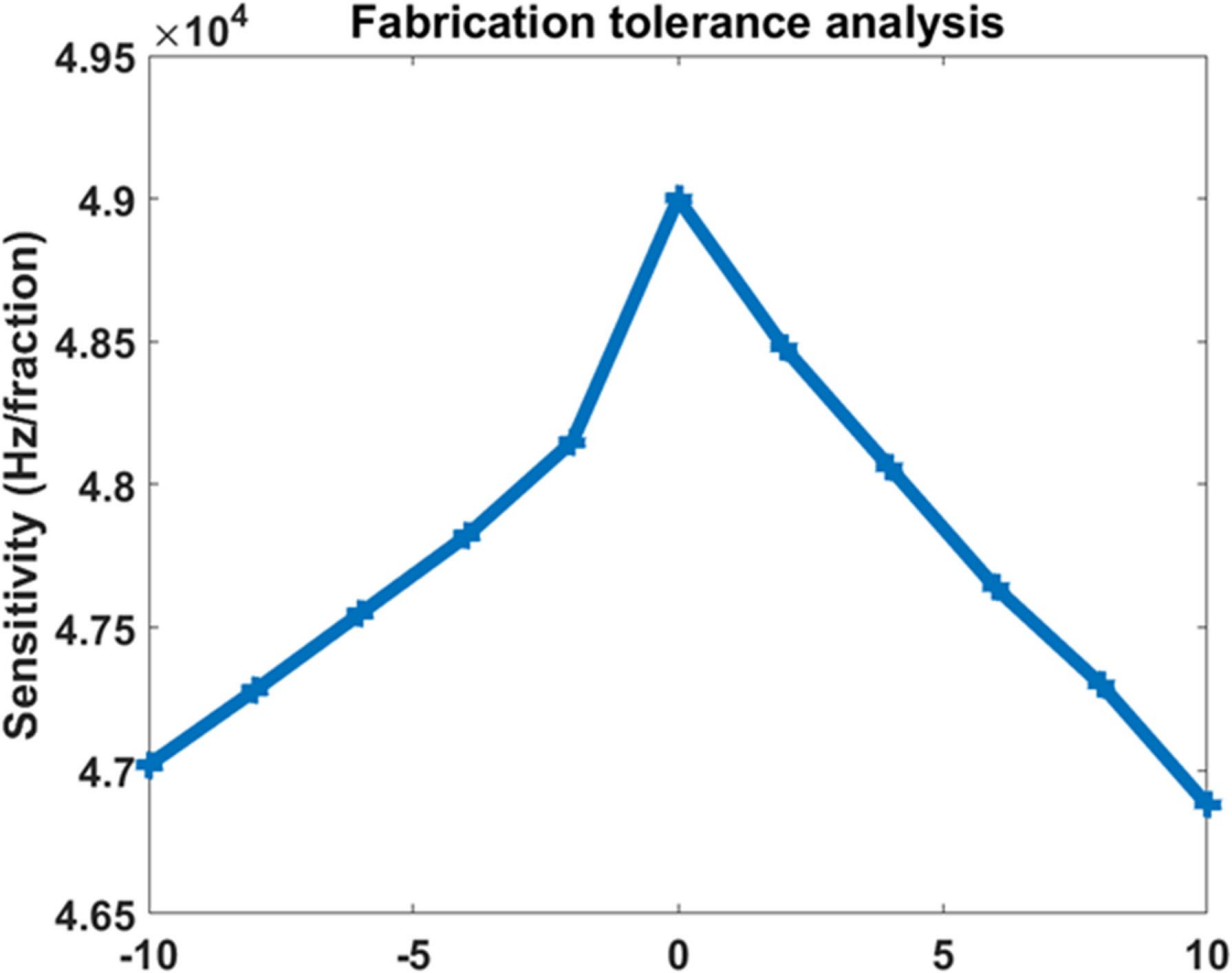
Fabrication tolerance analysis of the proposed phononic sensor under defect-layer thickness variationsfrom −10% to +10%, showing stable sensitivity around (4.70–4.90)×10^4^Hz.fraction^-1^ and confirming good robustness against fabrication imperfections.

Figure 8 presents the fabrication tolerance analysis performed by varying the defect-layer thickness from − 10% to + 10%. The sensitivity remains within a relatively narrow variation range around (4.70 − 4.90)×10^4^ Hz.fraction^− 1^ indicating strong structural robustness against fabrication imperfections. The maximum sensitivity occurs near the nominal design thickness, while moderate deviations produce only limited performance degradation. Such behavior confirms that the proposed sensor can tolerate realistic fabrication errors without significant sensing deterioration. Physically, the stability originates from the strong defect-mode confinement generated by the asymmetric coupled-cavity architecture, which prevents rapid resonance collapse under geometrical perturbations. The tolerance analysis therefore demonstrates the practical manufacturability of the proposed phononic sensor for experimental realization and real-world sensing applications.

**Table 1:** Qualitative comparison of cavity-design strategies in phononic crystal sensors.

Structure type	Resonance confinement	Sensitivity trend	Q-factor trend	Main limitation	Distinguishability	References
Conventional single-defect cavity	Strong localized confinement	Moderate	Relatively high	Limited analyte-mode interaction	Moderate	^4–8,17^
Symmetric coupled cavity	Balanced confinement	Improved	Moderate	Less tunable field distribution	High	^31–37^
Super-asymmetric coupled cavity (this work)	Asymmetry-controlled confinement	Highest in the present study: 4.90 × 10^4^ Hz.fraction^− 1^	335–1080	Concentration-dependent broadening	High	Present work

The first two rows summarize qualitative trends reported for previously studied defect-based and coupled-cavity phononic crystal sensing platforms, as indicated by the cited references. The proposed super-asymmetric coupled-cavity structure was evaluated quantitatively in the present work.

Table 1 summarizes the qualitative differences between conventional single-defect, symmetric coupled-cavity, and the proposed super-asymmetric coupled-cavity phononic sensor designs. The comparison is intended to clarify the design role of asymmetric coupling rather than to provide a direct numerical ranking against previously reported structures. Conventional defect cavities usually provide strong resonance confinement but may limit analyte-mode interaction, whereas symmetric coupled cavities can improve interaction while maintaining acceptable spectral sharpness. In the proposed super-asymmetric configuration, the unequal coupling layers modify the acoustic confinement around the liquid defect cavity, producing a clear concentration-dependent resonance shift with a sensitivity of 4.90 × 10^4^ Hz.fraction^− 1^ and a Q-factor range of 335–1080. Therefore, the main advantage of the proposed design is not simply a higher sensitivity value, but the controlled balance between resonance tunability, spectral distinguishability, and fabrication robustness.

**Table 2:** Comparison of phononic crystal-based biosensors in terms of sensitivity, Q-factor, and structural features.

Sensor type/structure	Sensitivity	Special feature	Q-factor	References
1D PhnCs	3816 Hz	Ethyl lactate and 2-butoxy ethanol	90,613	^31^
1D PhnCs	566 Hz/%	Membrane for Liquid	500	^32^
1D PhnCs	195 Hz	Sucrose detection	53,515	^33^
1DPhnCs	969.973 Hz	Glycine	–	^34^
PhnCs	12.54 Hz/%	Methanol	–	^35^
1D PhnCs	0.91 × 10^3^ Hz (ms^−1^)	Gasoline-ethanol	200	^36^
2D periodic structure	0.4 × 10^3^ Hz (ms^− 1^)	Propanol	600	^31^
1D PhnCs	4.9 × 10⁴ Hz⋅fraction^−1^	Super-asymmetric coupled cavity; ethanol–water mixture	335–1080	Proposed work

Sensitivity values are reported in different units depending on the sensing mechanism; therefore, the comparison should be interpreted qualitatively rather than as a direct numerical ranking.

Table 2 compares the proposed sensor with previously reported phononic crystal-based sensing configurations. Since the reported sensitivities are expressed in different units depending on the target analyte and sensing mechanism, the comparison is mainly intended to highlight the structural and performance trends rather than provide a direct numerical ranking. The proposed 1D super-asymmetric coupled-cavity sensor achieves a sensitivity of 4.90 × 10^4^ Hz.fraction^-1^ with a Q-factor ranging from 335 to 1080 across the ethanol–water concentration range. This indicates that the design maintains measurable resonance sharpness while providing a clear concentration-dependent frequency shift.

## Conclusion

In this study, a one-dimensional super-asymmetric coupled-cavity phononic crystal sensor was proposed and analyzed for ethanol–water concentration sensing. The introduction of asymmetric coupling cavities around the liquid defect region modified the acoustic localization behavior and enabled stronger concentration-dependent resonance tuning compared with conventional symmetric cavity configurations. The obtained transmission spectra demonstrated a clear and continuous defect-mode shift across the investigated concentration range while preserving spectrally distinguishable resonance peaks. The proposed structure achieved a sensitivity of approximately 4.90 × 10^4^ Hz.fraction^-1^ with excellent linearity (R^2^ = 0.999990), confirming the stability of the resonance evolution with concentration. In addition, the Q-factor analysis revealed that resonance confinement gradually decreases with increasing water fraction, indicating a practical trade-off between spectral sharpness and tunability. The transmission distribution analysis further confirmed the stable progression of the resonance mode without spectral overlap or abrupt distortion. Moreover, the fabrication tolerance investigation demonstrated that moderate defect-thickness deviations produce only limited sensitivity degradation, supporting the robustness of the proposed design against fabrication imperfections. Overall, the obtained results indicate that super-asymmetric acoustic confinement can provide an effective mechanism for enhancing resonance tunability in one-dimensional phononic sensing platforms. The proposed configuration may therefore offer potential for compact liquid-analysis and concentration-monitoring applications based on acoustic spectral detection.

## Data Availability

The data generated and analyzed during this study, including the numerical data underlying the figures, are available from the corresponding author upon reasonable request.
